# Publish or perish in paediatric ophthalmology and strabismus – where do we stand?

**DOI:** 10.1038/s41433-024-03013-4

**Published:** 2024-03-22

**Authors:** L. Solomon-Cohen, E. Mezer, T. Wygnanski-Jaffe

**Affiliations:** 1 Arrow Program for Medical Research Education, Sheba Medical Center, 5265601 Tel-Hashomer, Israel; 2 Faculty of Medicine, Tel-Aviv University, 6139001 Tel-Aviv, Israel; 3 Bruce Rappaport Faculty of Medicine, Technion – Israel Institute of Technology, Haifa, Israel; 4 Department of Ophthalmology, Ruth Rappaport Children’s Hospital, Rambam Health Care Campus, Haifa, Israel; 5 Pediatric Ophthalmology Unit. The Goldschleger Eye Institute - Sheba Medical Center, Tel HaShomer, Ramat-Gan, Israel

**Keywords:** Health care, Paediatrics

## To the Editor:

The “publish or perish” phenomenon is a reality in medical research, where continuous publication is essential for career advancement and overall success. We investigated the impact of this phenomenon in paediatric ophthalmology and strabismus (POS) for 2022 in 12 leading journals (Supplementary-e-table-1) and identified the ten most prolific authors who had implemented a previous methodology [1]. We excluded editorials, letters, or comments.

Our search yielded 343 articles including 1786 authors (average 1.19 ± 0.79, median 1, range 1–15 articles per author). Forty percent of the ten most prolific authors were women, twice as many compared to that in general ophthalmology (20%) [1]. The narrowing gender gap in POS was previously reported in another publication [2].

A full PubMed search revealed an average of 17.4 ± 9.9 (median 14.5) articles, indicating a vast difference from the remaining POS authors. Articles were published in journals with an average impact factor of 5.9 ± 5.8 (median 3.9). This diversity indicates a wide spectrum of journals, not limited to top-tier ophthalmology publications.

These prolific authors were in a first-author position in 14.16% ± 18.9% (median 7.7%) of the articles, a middle position in 52.2% ± 27.8% (median 48.4%), and the last position in 33.7% ± 22.3% (median 32.05%) (Table 1). The fact that half of the contributions were in middle positions may imply the significant role of prolific authors, even when they are not the principal authors, presumably due to their experience level and reputation.

**Table 1:** The most prolific authors of scientific papers and their positions in the byline.

Author number	Number of articles in selected journals	Total number of articles	First position	Middle position	Last position	First position (%)	Middle position (%)	Last position (%)
1	15	23	0	10	13	0%	43%	57%
2	12	13	2	7	4	15.4%	53.8%	30.8%
3	11	17	0	6	11	0%	35.3%	64.7%
4	9	13	4	3	6	30.8%	23.08%	46.2%
5	8	17	3	12	2	17.7%	70.6%	11.8%
6	8	15	9	2	4	60%	13.3%	26.7%
7	8	14	1	5	8	7.1%	35.7%	57.1%
8	8	12	1	7	4	8.3%	58.3%	33.3%
9	7	43	1	38	4	2.3%	88.4%	9.3%
10	7	7	0	7	0	0%	100%	0%
Mean	9.3	17.4	2.1	9.7	5.6	14.16%	52.2%	33.7%
SD	2.6	9.9	2.8	10.4	4	18.9%	27.8%	22.3%
Median	8	14.5	1	7	4	7.7%	48.4%	32.05%

Studies led by research groups comprised 26.4 ± 13.6% (median 26.1%). Randomized controlled trials (RCTs) comprised 16.5 ± 10.4% (median 14.1%) (Table 2). The average Oxford Centre for Evidence-Based Medicine scheme rank [3] for the top 10 prolific authors was 2.6 ± 0.5 (median 2.8). A median of 18.8% achieved the highest rank (level 1), denoting good quality RCT or systemic reviews of randomized trials, compared with none of the articles in general ophthalmology [1] (Supplementary-e-Table-2). Furthermore, in a study that examined multiple fields of medicine, including epilepsy, rheumatoid arthritis, renal transplantation, and liver transplantation, only 1.6% of papers published by the 10 most prolific authors over 5 years were systematic reviews, whereas 22.3% were clinical trials [4]. Note that the articles did not provide information on the quality of the clinical trials; therefore, their association with the Oxford scheme ranking could not be determined. This nuanced contrast highlights the possibly better maintenance of high research quality by prolific authors in the specialized area of POS compared with general ophthalmology or other fields of medicine.

**Table 2:** The writing characteristics of the most prolific authors.

Author number	Number of articles	Impact factor (mean)	Impact factor (SD)	Impact factor (median)	Part of a research group (%)	Is a RCT (%)
1	23	5.8	4.4	4.4	34.8%	30.4%
2	13	5.4	3.9	3.5	46.1%	38.5%
3	17	6	6.3	1.8	17.6%	17.6%
4	13	6.4	5.2	5.5	15.4%	15.4%
5	17	8.2	5.2	8.3	23.5%	5.9%
6	15	6.1	3.8	5.5	33.3%	13.3%
7	14	6.1	3.7	5.5	28.6%	7.1%
8	12	4.9	2.9	4.1	41.7%	8.3%
9	43	4.8	7.4	3.1	23.3%	14%
10	7	7	5.7	1.9	0%	14.3%
Mean	17.4	5.9			26.4%	16.5%
SD	9.9	5.8			13.6%	10.4%
Median	14.5	3.9			26.1%	14.1

Interestingly, in 2022 the top 10 prolific authors in POS published 14.5 times as many articles as all POS authors combined. They published higher-ranking article types in journals with a higher impact factor compared with general ophthalmology or other fields of medicine. However, they were not the principal authors in more than half of the publications. The gender gap was smaller by half, compared with general ophthalmology, adding to the trend previously reported in POS [2]. These observations on this unique group may inspire others to excel.

### Supplementary information

Supplementary e table 1

Supplementary e table 2
