# Supplementary material for: Publish or perish in paediatric ophthalmology and strabismus – where do we stand?
Source: Eye (Lond). 2024 Mar 22;38(9):1774–5. doi: 10.1038/s41433-024-03013-4 (PMC11156892; doi:10.1038/s41433-024-03013-4)
Supplement: Supplementary file 1 — Supplementary e table 1 [file 41433_2024_3013_MOESM1_ESM.docx]

Supplementary-e-table-1 – list of leading peer reviewed journal used to rank the 10 highest prolific authors in pediatric ophthalmology and strabismus for 2022

| Chosen journals: |
| --- |
| Ophthalmology |
| JAMA Ophthalmology |
| American Journal of Ophthalmology |
| British Journal of Ophthalmology |
| Eye |
| Acta Ophthalmologica |
| Clinical & Experimental Ophthalmology |
| Grafe’s Archives Clinical Experimental Ophthalmology |
| European Journal of Ophthalmology |
| Journal of AAPOS |
| Journal of POS |
| Strabismus |
