# Supplementary material for: Publish or perish in paediatric ophthalmology and strabismus – where do we stand?
Source: Eye (Lond). 2024 Mar 22;38(9):1774–5. doi: 10.1038/s41433-024-03013-4 (PMC11156892; doi:10.1038/s41433-024-03013-4)
Supplement: Supplementary file 2 — Supplementary e table 2 [file 41433_2024_3013_MOESM2_ESM.docx]

Supplementary-e-table-2 – Classification of each author’s articles according to the Oxford Centre for Evidence-based Medicine ranking:

| Author  number | Number of articles | No. of level 1 articles  (%) | No. of level 2 articles  (%) | No. of level 3 articles  (%) | No. of level 4 articles  (%) | No. of level 5 articles  (%) | Oxford scheme  rank (Mean) | Oxford  Scheme  rank  (SD) | Oxford  Scheme  rank  (Median) |
| --- | --- | --- | --- | --- | --- | --- | --- | --- | --- |
| 1 | 23 | 8 (34.8%) | 10 (43.5%) | 1 (4.3%) | 4 (17.4%) | 0  (0%) | 2 | 1.1 | 2 |
| 2 | 13 | 8 (61.5%) | 3 (23.1%) | 1 (7.7%) | 1  (7.7%) | 0  (0%) | 1.6 | 1 | 1 |
| 3 | 17 | 3 (17.6%) | 9 (52.9%) | 1 (5.9%) | 4 (23.5%) | 0  (0%) | 2.4 | 1.1 | 2 |
| 4 | 13 | 2 (15.4%) | 4  (30.8%) | 1 (7.7%) | 5  (38.5%) | 1 (7.7%) | 2.9 | 1.3 | 3 |
| 5 | 17 | 2 (11.8%) | 7 (41.2%) | 0  (0%) | 5 (29.4%) | 3 (17.6%) | 3.2 | 1.5 | 3 |
| 6 | 15 | 3  (20%) | 4 (26.7%) | 0  (0%) | 8 (53.3%) | 0  (0%) | 2.9 | 1.3 | 4 |
| 7 | 14 | 1 (7.1%) | 3 (21.4%) | 0  (0%) | 9 (64.3%) | 1 (7.1%) | 3.4 | 1.2 | 4 |
| 8 | 12 | 4 (33.3%) | 2 (16.7%) | 1 (8.3%) | 4 (33.3%) | 1 (8.3%) | 2.7 | 1.5 | 2.5 |
| 9 | 43 | 9 (20.9%) | 13  (30.2%) | 0  (0%) | 17 (39.5%) | 4 (9.3%) | 2.9 | 1.4 | 2 |
| 10 | 7 | 1 (14.3%) | 4 (57.1%) | 1 (14.3%) | 1 (14.3%) | 0  (0%) | 2.3 | 1 | 2 |
| Mean | 17.4 | 4.1 (23.7%) | 5.9  (34.4%) | 0.6 (4.8%) | 5.8 (32.1%) | 1  (5%) | 2.6 | 1.2 | 2.6 |
| SD | 9.9 | 3.1 (15.9%) | 3.7  (13.7%) | 0.5 (4.9%) | 4.7 (17.6%) | 1.4 (6%) | 0.5 | 0.2 | 1 |
| Median | 14.5 | 3 (18.8%) | 4 (30.5%) | 1 (5.1%) | 4.5 (31.7%) | 0.5 (3.6%) | 2.8 | 1.2 | 2.3 |
